# Central Nervous System‐Active Medications and Risk of Hospital Readmission in Older Multimorbid Adults

**DOI:** 10.1111/jgs.70049

**Published:** 2025-08-29

**Authors:** Mirah J. Stuber, Lara A. Brockhus, Anne Spinewine, Denis O'Mahony, Emma Jennings, Olivia Dalleur, Wilma Knol, Huiberdina L. Koek, Stéphanie Baggio, Nicolas Rodondi, Carole E. Aubert

**Affiliations:** ^1^ Department of General Internal Medicine Inselspital, Bern University Hospital, University of Bern Bern Switzerland; ^2^ Institute of Primary Health Care (BIHAM), University of Bern Bern Switzerland; ^3^ Clinical Pharmacy and Pharmacoepidemiology Research Group, Louvain Drug Research Institute Université Catholique de Louvain Belgium; ^4^ Department of Pharmacy CHU UCL Namur Yvoir Belgium; ^5^ Department of Medicine, School of Medicine University College Cork Cork Republic of Ireland; ^6^ Pharmacy Cliniques Universitaires Saint‐Luc Brussels Belgium; ^7^ Department of Geriatric Medicine and Expertise Centre Pharmacotherapy in Old Persons University Medical Center Utrecht, Utrecht University Utrecht the Netherlands; ^8^ Department of Geriatric Medicine University Medical Center Utrecht, Utrecht University Utrecht the Netherlands; ^9^ Laboratory of Population Health (#PopHealthLab), University of Fribourg Fribourg Switzerland

**Keywords:** hospital readmission, multimorbid, polypharmacy, psychotropic medications

## Abstract

**Background:**

Polypharmacy is associated with adverse outcomes, particularly in older multimorbid adults. However, little is known about the negative outcomes associated with multiple central nervous system (CNS)‐active medications that are commonly prescribed to these patients.

**Objective:**

To assess the association between the number of CNS‐active medications at discharge and the risk of 1‐year all‐cause hospital readmission, drug‐related hospital readmission (DRA), death, quality of life (QoL) and functional status in older multimorbid adults.

**Methods:**

Among 2008 older multimorbid inpatients with polypharmacy, we assessed the association between the number of CNS‐active medications and 1‐year all‐cause hospital readmission, DRA, and death by Cox proportional hazard models. We further assessed the association of the number of CNS‐active medications with QoL (measured with EQ‐5D‐VAS) and functional status (measured with Barthel Index) using binary and quantile regression models. Analyses were adjusted for age, sex, discharge location, Charlson Comorbidity Index, depression/anxiety, and randomization arm. Additional sensitivity analyses were adjusted for the number of non‐CNS active medications, neurological and psychiatric comorbidities, alcohol or tobacco use, education level, and living arrangements.

**Results:**

The risk of all‐cause hospital readmission and DRA increased by 7% with each additional CNS‐active medication (multivariable‐adjusted hazard ratio (HR) 1.07 (95% confidence interval 1.03 to 1.12) for all‐cause hospital readmission and 1.07 (1.01 to 1.14) for DRA). HR for death was 1.14 (1.07 to 1.23) for each additional CNS‐active medication. The mean differences in EQ‐5D‐VAS and Barthel Index after 1 year were −2.13 (−2.82 to −1.44) and −1.6 (−2.16 to −1.04) respectively, per additional CNS‐active medication.

**Conclusion:**

The presence of CNS‐active medications at discharge is associated with a higher risk for 1‐year all‐cause hospital readmission and DRA in older multimorbid adults with polypharmacy. Additionally, CNS‐active medications were associated with lower QoL and functional status.

**Trail Registration:** ClinicalTrials.gov NCT02986425

**Summary**

- Key points
  ○ Each additional concomitant CNS‐active medication at discharge was associated with 7% increased risk of all‐cause hospital readmission.
  ○ The number of CNS‐active medications was associated with lower quality of life and reduced functional status.
- Why does this paper matter?
  ○ CNS‐active medications are frequently prescribed in multimorbid patients with polypharmacy. Our findings highlight that prescription of these medications should be carefully assessed.

## 1 Introduction

The older population is growing rapidly, with the number of people aged 65 years and older projected to more than double between 2019 and 2050 worldwide [1]. Multimorbidity increases with age and is estimated to affect at least 70% of people aged ≥ 65 years [2, 3, 4, 5]. It is associated with higher mortality, hospital readmission rates, and health care utilization [2, 3, 4, 5]. In addition, multimorbidity often results in the prescription of multiple concomitant medications, which may well be indicated individually for the various conditions, but in combination entails a higher risk of iatrogenic morbidity. The daily intake of ≥ 5 long‐term medications, referred to as polypharmacy, leads to an increased risk of inappropriate prescribing, which in turn is associated with important negative outcomes such as drug‐related hospital readmissions (DRAs), falls, and increased mortality [6].

In patients with polypharmacy, central nervous system (CNS)‐active medications are among the most commonly prescribed medications, with many of them prescribed inappropriately (longer than needed, without clear indication) [7, 8, 9, 10, 11]. This is partly due to the fact that psychiatric and neurological disorders affect up to 30% of people aged ≥ 65 years [12, 13]. The high prevalence of CNS‐active medications of around 60% in older adults with polypharmacy carries associated iatrogenic risks [9]. CNS‐active medications are among the drug classes most affected by age‐related pharmacodynamic alterations, resulting in increased susceptibility among older adults to several adverse effects [14]. In addition, with aging, we observe a higher morbidity and more medications (and consequent risks of drug–drug and drug‐disease interactions), as well as a cumulative effect of medication use and more frequent acute illnesses, all of which can increase this susceptibility. This is reflected, for example, in the well‐known negative outcomes associated with benzodiazepines and antidepressants in older adults [15, 16]. While the long‐term use of benzodiazepines comes with an increased risk of falls and fractures, functional decline and death, selective serotonin reuptake inhibitors are associated with falls, fractures, and hyponatremia, all leading to increased risk of hospitalization [17, 18, 19, 20]. Furthermore, about 20% of DRAs are primarily related to CNS‐active medications [21]. Consequently, several CNS‐active medications have been incorporated into the American Geriatric Society (AGS) Beers criteria and the STOPP/START criteria in Europe [22, 23].

Despite these being well‐known problems with CNS‐active medications in older people, relatively little is known about the negative outcomes associated with the concomitant use of multiple CNS‐active co‐medications. In particular, few studies have investigated the association of multiple CNS‐active medications and the risk of hospital readmission. Previous studies on this topic have been limited by retrospective design and were often restricted to specific patient groups, such as patients with dementia [24, 25, 26]. The aim of our study was to examine the relationship between the number of CNS‐active medications and important clinical outcomes, namely hospital readmission, DRA, and all‐cause mortality in older adults with multimorbidity and polypharmacy. We also assessed patient‐reported outcomes, namely quality of life (QoL) and functional status and their relationship with the use of CNS‐active medication. Regarding the same outcomes, we furthermore aimed to assess adverse interaction effects between different combinations of CNS‐active medications.

## 2 Methods

### 2.1 Study Design

For this study, we undertook an analysis of prospectively collected data from the multinational OPERAM (“Optimizing Therapy to Prevent Avoidable Hospital Admissions in Multimorbid Older Adults”) trial, whose design and results have been described in detail previously [11, 27]. Briefly, this multinational cluster randomized controlled trial was designed to test a software intervention designed to optimize prescribed medication in older hospitalized patients with multimorbidity and polypharmacy. Specifically, the trial tested the ability of the intervention to prevent DRAs by comparing usual medication assessment with the pharmacotherapy optimization intervention applied by an interprofessional team of a trained pharmacists and physicians [27].

### 2.2 Patients and Setting

The OPERAM trial included 2008 multimorbid (≥ 3 chronic conditions) patients aged 70 years or older with polypharmacy (≥ 5 chronic medications) admitted to medical or surgical wards at 4 large university medical centers in four European countries (Belgium, Ireland, Netherlands and Switzerland). To increase external validity, few exclusion criteria were applied, that is, planned transfer to palliative care within 24 h of readmission, report of a structured medication review by a clinician within 2 months prior to readmission, and inability to provide written informed consent by the patient or a proxy. Patients were enrolled from December 2016 to October 2018 and followed up over 12 months after enrolment. Patients were screened for eligibility at hospital admission and enrolled during hospitalization (follow‐up starting on date of enrolment). Duration of index hospitalizations varied between patients. Follow‐up telephone interviews were performed by blinded researchers at 2, 6, and 12 months after randomization.

### 2.3 Exposure

The exposure of interest was the number of CNS‐active medications at discharge after the index hospitalization during which patients were enrolled in the OPERAM trial. CNS‐active medications were identified by the Anatomical Therapeutic Chemical (ATC) classification codes [28] and defined as: opioids, antimigraine preparations, antiepileptic medications, antiparkinsonian medications, psycholeptics (antipsychotics, anxiolytics, hypnotics and sedatives), antidepressants, antidementia medications, parasympathomimetics, medications used in addictive disorders, and antivertigo preparations (see eTable S2 for complete list of ATC codes). The definition of CNS‐active medications was based on the list of medications acting on the central nervous system according to the ATC index of the WHO Collaborating Centre for Drug Statistics Methodology.

### 2.4 Outcomes and Potential Confounders

The clinical outcomes of interest were the first all‐cause hospital readmission, first DRA, and death between exposure and 12 months after study enrolment. Hospital readmissions were ascertained during follow‐up telephone interviews at 2, 6, and 12 months after enrolment by blinded researchers who asked the patient or a proxy about any hospital stay since the last hospitalization. An independent, blinded adjudication committee assessed drug relatedness of each hospital readmission using a standardized adjudication guideline [29]. According to this guideline, DRAs were defined as diagnoses that were caused by an adverse drug event related to over‐, under‐, or misuse of medications (e.g., falls, hyperkalaemia, acute renal impairment) [29].

Patient‐reported outcomes included QoL and functional status in terms of independence in activities of daily living (ADLs). QoL was assessed with the visual analogue scale component of the European quality of life‐5 dimensions (EQ‐5D) questionnaire (EQ‐5D‐VAS, EuroQol Group. EQ‐5D is a trade mark of the EuroQol Group) [30] and functional status was assessed using the Barthel Index basic ADL questionnaire [31]. Both QoL and ADLs were assessed at study enrolment and 12‐month follow‐up. The Minimal Clinically Important Difference lies between 7 and 9 points for the Barthel Index and 7–10 points for the EQ‐5D‐VAS [32, 33, 34]. EQ‐5D‐VAS and Barthel Index were both measured on a scale from 0 to 100.

Potential confounders were collected at the time of enrollment, except for ICD‐coded diagnoses, which were extracted from the discharge letter of the index hospitalization. These ICD‐coded diagnoses included all diagnoses from before the hospitalization until discharge. We identified potential confounders based on clinical plausibility and used directed acyclic graphs to identify confounding variables, which might influence the number of CNS‐active medications as well as the outcomes and thus require conditioning (eFigure S1) [35]. Based on this assessment, we considered the following variables as potential confounders for hospital readmission: age, sex, discharge destination (discharge from cluster unit to home, nursing home, external rehabilitation, other hospital, home of relatives, other ward or intermediate care unit/intensive care unit), Charlson Comorbidity Index (calculated with the ICD‐10 coding algorithm of Quan et al.) [36, 37], presence of depression or anxiety, and randomization arm.

### 2.5 Statistical Analyses

We assessed the association of the number of CNS‐active medications at discharge with the first all‐cause hospital readmission, the first DRA, and death within 12 months by Cox proportional hazard models (the three outcomes were assessed separately). We accounted for the potential overestimation of event hazards by Cox proportional hazard models in the presence of competing risks. To address this, we performed an additional analysis using extensions of the Fine‐Gray proportional hazards model, treating death as a competing event, to calculate subdistribution hazard ratios (SHRs) [38]. For all Cox proportional hazard models, we statistically and graphically tested the proportionality assumption using Schoenfeld and scaled Schoenfeld residuals. We adjusted the models for the confounding baseline variables as mentioned in the section above. The variables were tested for collinearity by checking covariance matrices.

Variables tested for interaction were (1) age and number of CNS‐active medications (since older people are more susceptible to adverse effects and medication interactions) [14] and (2) Charlson Comorbidity Index and number of CNS‐active medications (since multimorbidity per se is known to affect pharmacokinetics and pharmacodynamics) [39]. To evaluate the interaction between different CNS‐active medication classes regarding the hazard of first all‐cause hospital readmission after index hospitalization, we introduced interaction terms for medication combinations that are clinically known for their interaction risk: opioids and hypnotics/sedatives, opioids and anxiolytics, hypnotics and antidepressants, and antipsychotics and antidepressants [40, 41, 42].

The duration (time at risk) was calculated in days from the time of discharge from the index hospitalization (time of exposure) to the date of first all‐cause hospital readmission, first DRA, death or end of follow‐up, respectively. For the analyses on the outcome of first all‐cause hospital readmission and first DRA, participant data were right censored if follow‐up was longer than 12 months, if they were lost to follow‐up, or in case of death. For the outcome of first all‐cause hospital readmission, we conducted prespecified subgroup analyses according to age (< 80 vs. ≥ 80 years) and presence/absence of dementia (yes/no). We did not perform subgroup analyses for the other outcomes, because we would have lacked power. We performed sensitivity analyses for various factors: number of non‐CNS active medications, neurological and psychiatric comorbidities (dementia, substance use disorder, psychosis and pain), alcohol and tobacco use, education level and living arrangement added as additional confounders in the main models. We conducted a further sensitivity analysis for chronic use of CNS‐active medication by censoring all patients that changed the number of CNS‐active at any of the follow‐ups (2, 6, 12 months). Furthermore, we treated death as a competing risk with a sub‐distribution hazard ratio (SHR) in analysis on time to first all‐cause hospital readmission or first DRA.

We assessed the association of the number of CNS‐active medications with functional status and QoL at 12 months after enrolment. The assumptions for linear regression were met for the EQ‐5D‐VAS, but not for the Barthel Index. We therefore used a linear regression for QoL and a logistic regression for the Barthel index (cut‐off at the median: score of 95) to estimate a Risk Ratio and an Adjusted Risk Ratio. Additionally, we conducted a Quantile Regression Model to estimate coefficients for an assessment of minimal clinically important changes (25th, 50th, 75th percentile).

Statistics were reported with their respective 95% confidence intervals (CI) and (two‐sided) *p* values.

Stata version 16.0 software (Stata Corporation, College Station, Tex) was used to perform the statistical analyses [43].

## 3 Results

Among the 2008 participants of the OPERAM trial, the median age was 79 years (interquartile range IQR 74–84 years) and 898 participants (44.7%) were female. Ten (0.5%) participants were lost to follow‐up, 118 (5.9%) withdrew from the trial, 2 follow‐ups were not completed, and 385 (19.2%) died (318 participants) within 12 months of enrolment (eFigure S2). Participants' median number of comorbidities was 11 (IQR 8–16), 930 (46.3%) participants were aged ≥ 80 years and 148 (7.4%) had dementia at baseline. Baseline characteristics of participants at index hospitalization are presented in Table 1. The median number of CNS‐active drugs was 1 (IQR 0–2) and 1141 (56.8%) participants were prescribed at least one CNS‐active drug at discharge. Distribution of the number of patients in relation to the number of CNS medications at discharge is displayed in eFigure S3. Psycholeptic drugs, opioids, and antidepressants were the most frequently prescribed CNS‐active drugs (prescribed in 538 (26.8%), 427 (21.3%), and 421 (21.0%) participants, respectively) (Table 1).

**TABLE 1:** Baseline characteristics of patients.

Characteristics	*N* = 2008
Age at enrolment, years, median (interquartile range)	79 (74–84)
Age ≥ 80 years, *N* (%)	930 (46.3)
Female, *N* (%)	898 (44.7)
Site	
Bern, Switzerland *N* (%)	822 (40.9)
Cork, Ireland *N* (%)	346 (17.2)
Louvain, Belgium *N* (%)	388 (19.3)
Utrecht, Netherlands *N* (%)	452 (22.5)
No. of comorbidities, median (interquartile range)	11 (8–16)
Quality of life, median (interquartile range)^a^	60 (50–73)
Activities of daily living, median (interquartile range)^b^	90 (75–100)
Depression or anxiety, *N* (%)	151 (7.5)
Dementia, *N* (%)	148 (7.4)
No. of non‐CNS‐active medications^c^, median (interquartile range)	10 (8–13)
At least one CNS‐active medication^c^, *N* (%)	1141 (56.8)
No. of CNS‐active medications^c^, median (interquartile range)	1 (0–2)
Patients with ≥ 1 CNS‐active medication(s), *N* (%)	1141 (56.8)
Patients with ≥ 1 opioid(s), *N* (%)	427 (21.3)
Patients with ≥ 1 antiepileptic(s), *N* (%)	297 (14.8)
Patients with ≥ 1 antiparkinson medication(s), *N* (%)	127 (6.3)
Patients with ≥ 1 antipsychotic(s), *N* (%)	130 (6.5)
Patients with ≥ 1 anxiolytic(s), *N* (%)	211 (10.5)
Patients with ≥ 1 hypnotic(s) and sedative(s), *N* (%)	290 (14.4)
Patients with ≥ 1 antidepressant(s), *N* (%)	421 (21.0)
Patients with ≥ 1 anti‐dementia medication(s), *N* (%)	38 (1.9)

^a^ Measured by visual analogue scale, second part of European quality of life‐5 dimensions questionnaire (EQ‐VAS). Values range from 0 to 100. Higher values indicate higher quality of life.

^b^ Measured using the Barthel Index. Values range from 0 to 100. Higher values indicate higher functional independence.

^c^ Including opioids, antimigraine preparations, antiepileptic medications, antiparkinson medications, psycholeptics (antipsychotics, anxiolytics, hypnotics and sedatives), antidepressants, antidementia medications, parasympathomimetics, medications used in addictive disorders, and antivertigo preparations. See eTable S1 for the full list of ATC codes.

### 3.1 All‐Cause Hospital Readmission, Drug‐Related Hospital Readmission, and Death

**FIGURE 1:**
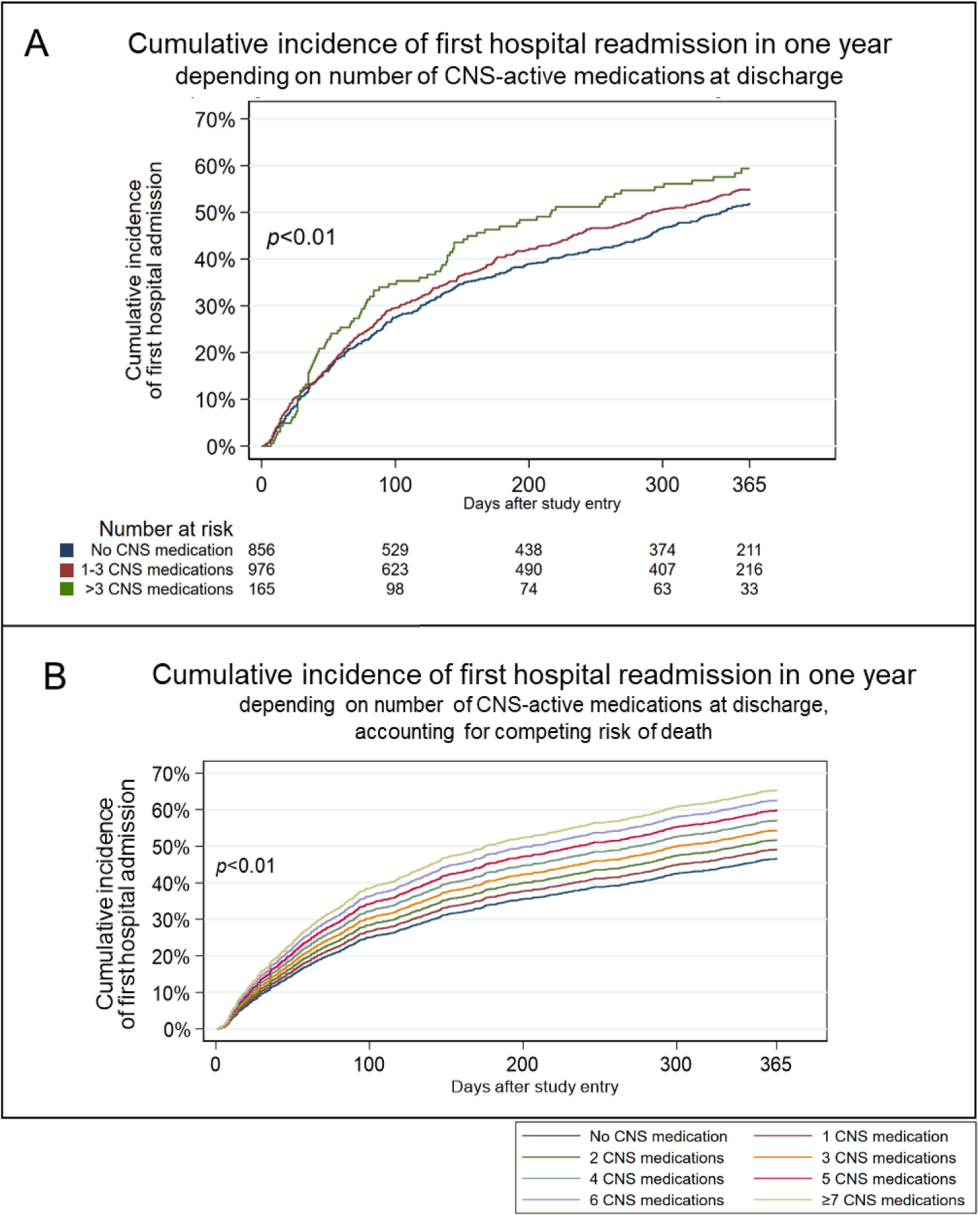
(A) Cumulative incidence of first all‐cause hospital readmission displayed by strata of patients with no CNS‐active medication, with 1–3 CNS‐active medications, and with > 3 CNS‐active medications. Curve truncated at 365 days. *p* value from Wald statistic. (B) Cumulative incidence of first all‐cause hospital admission displayed by different number of concomitant CNS‐active medications. Curve truncated at 365 days. *p* value from Wald statistic.

Overall, 963 (48%) participants had at least one hospital readmission during the observed follow‐up time window of 12 months. Of these patients, almost 50% (444 participants, 22.1% of all) were readmitted due to DRA in whom the median time to first hospital readmission was 84 days. We found no significant interaction between patients' age and number of CNS‐active medications (HR 1.00 (95% CI 0.99 to 1.00)) or between Charlson Comorbidity Index and number of CNS‐active medications (HR 1.02 (95% CI 1.00 to 1.04)). The adjusted hazard ratio for the first all‐cause hospital readmission within 12 months was 1.07 (95% CI 1.03 to 1.12) for each additional CNS‐active medication (Table 2). When treating death as a competing risk, we found a sub‐distribution hazard ratio (SHR) of 1.08 (95% CI 1.03 to 1.12) (Table 2). A log likelihood test supported the model, which assumed a linear association between the number of CNS‐active medications and first all‐cause hospital readmission, suggesting a dose–response relationship. Figure 1 graphically summarizes the cumulative incidence of first all‐cause hospital readmission depending on the number of CNS‐active medications. For a clearer presentation, Figure 1A displays the result from the Cox proportional hazards model stratified into three larger groups (participants with 0, 1–3, and > 3 CNS‐active medications) and Figure 1B displays the results from the Fine‐Gray proportional hazards model stratified into eight groups (participants with 0, 1, …, 6, ≥ 7 CNS‐active medications).

**TABLE 2:** Association between the number of CNS‐active medications and all‐cause readmission, DRA, death, quality of life, and activities of daily living.

Main outcomes					
	No. (%) of events overall	Adjusted hazard ratio (95% CI)^a^ for each additional CNS‐active medication	*p*	Adjusted SHR (95% CI)^b^ for each additional CNS‐active medication	*p*
First all‐cause hospital readmission	963 (48.0%)	1.07 (1.03 to 1.12)	< 0.01	1.08 (1.03 to 1.12)	< 0.01
First drug‐related hospital readmission	444 (22.1%)	1.07 (1.01 to 1.14)	0.03	1.07 (1.00 to 1.14)	0.03
Death	318 (16.6%)	1.14 (1.07 to 1.23)	< 0.01	NA	NA

Patient‐reported outcomes					
	No. (%)^c^	Adjusted relative risk (95% CI)^d^ for each additional CNS‐active medication	*p*	Adjusted difference (95% CI)^e^ for each additional CNS‐active medication	*p*
Quality of life^f^	1360 (67.7%)	0.91 (0.88 to 0.95)	< 0.01	−2.13 (−2.82 to −1.44)	< 0.01
Activities of daily living (ADLs)^g^	1378 (68.6%)	0.96 (0.95 to 0.98)	< 0.01	−1.60 (−2.16 to −1.04)	< 0.01

Abbreviations: CI, confidence interval; SD, standard deviation; SHR, subdistribution hazard ratio.

^a^ Adjusted for age (at baseline), sex, discharge location, Charlson Comorbidity Index (at baseline), presence of depression or anxiety (at baseline) and randomization arm. Hazard ratios > 1 indicate more events with increasing numbers of CNS‐active medications.

^b^ Subdistribution hazard ratio according to the Fine‐Gray model; adjusted for age (at baseline), sex, discharge location, Charlson Comorbidity Index (at baseline), presence of depression or anxiety (at baseline) and randomization arm. Hazard ratios > 1 indicate more events with an increasing number of CNS‐active medications.

^c^ Number of participants with outcome at 12 months available; non‐available data at 12 months was mainly due to death.

^d^ Adjusted risk ratio using a binary regression model with a cut‐off at the median.

^e^ Adjusted difference in coefficients using a quantile regression model.

^f^ Measured by visual analogue scale, second part of European quality of life‐5 dimensions questionnaire (EQ‐5D‐VAS). Values range from 0 to 100. Higher values indicate higher quality of life.

^g^ Measured using the Barthel Index. Values range from 0 to 100. Higher values indicate higher functional independence.

We obtained similar findings in prespecified subgroup analyses (presence of dementia and age ≥ 80 years) without significant interactions between age and number of CNS‐active medications nor between presence of dementia and number of CNS‐active medications (eFigure S4).

At least one DRA during the past 12 months was observed in 444 (22.1%) of the participants. The median time to first DRA was 107 days. The hazard ratio for first DRA was 1.07 (95% CI 1.01 to 1.14), with similar results in the sensitivity analysis accounting for the competing risk of death (SHR 1.07; 95% CI 1.00 to 1.14).

Death within 12 months occurred in 318 (16.6%) of patients with a median time to death of 76 days. The hazard ratio for death was 1.14 for each additional CNS‐active medication (95% CI 1.07 to 1.23).

The result of the sensitivity analyses adjusting for non‐CNS active medications, neurological and psychiatric comorbidities, alcohol and tobacco use, education level, and living arrangement remained similar to those of the main analyses for first all‐cause readmission, first DRA, and death. Patients that did not deviate from their number of CNS‐active medications at discharge within the 12 months follow‐up period showed a higher association with first all‐cause readmission (HR 1.21, 95% CI 1.15 to 1.27) first DRA (HR 1.24, 95% CI 1.15 to 1.34) and death (HR 1.20, 95% CI 1.11 to 1.30) (eTable S2).

We did not find statistically significant interactions between opioids and hypnotics/sedatives, between opioids and anxiolytics, between hypnotics and antidepressants, or between antipsychotics and antidepressants (eTable S3).

### 3.2 Patient‐Reported Outcomes

In multivariable analyses, the number of CNS‐active medications was associated with a lower QoL score and a lower probability of having a Barthel Index (functional status) score higher than 95 after 12 months. The adjusted mean difference was −2.13 points (95% CI −2.82 to −1.44) in the EQ‐5D‐VAS and −1.6 (95% CI −2.16 to −1.04) in the Barthel Index for each additional CNS‐active medication, respectively (Table 2). Patients with lower functional status showed a higher probability of better functional status with each additional CNS‐active medication, with an adjusted mean difference of −2.80 points (95% CI −3.84 to −1.76). For patients with higher functional status, the benefit went to null (−0 points (95% CI −0.04 to 0.04)) (Table 2).

## 4 Discussion

In this analysis of older patients with multimorbidity and polypharmacy of the OPERAM trial, approximately 60% of participants were prescribed at least one CNS‐active medication. Almost half of these patients experienced at least one hospital readmission within 12 months. We observed an increase in the risk of all‐cause hospital readmission and DRA of 7% with each additional CNS‐active medication at discharge. The association regarding all‐cause hospital readmissions followed a dose–response relationship and was not explained by potential confounders such as the number of comorbidities or age. We found an association between all‐cause mortality and the number of CNS‐active medications at discharge, as well as an association with a lower QoL score and poorer functional status. For QoL, this association was observed to be clinically relevant beginning at 4 CNS‐active medications and for the Barthel Index at more than 5 CNS‐active medications (Minimal Clinically Important Difference 7–10 and 7–9 points, respectively) [32, 33, 34].

Published evidence on the risks associated with intake of multiple CNS‐active medications is scarce. Regarding the outcome of all‐cause hospital readmission, our findings are consistent with those of a large register‐based case–control study from Sweden involving an older general population [26]. The authors found a 27% increased risk of hospitalization during 1 year in patients taking four psychotropic medications concomitantly compared to patients taking no psychotropic medications. In addition, this study observed a linear association between the number of psychotropic medications and the risk of hospital readmission. The study is comparable to ours in terms of the population studied (older patients), but differs in terms of design, quality of data collection, and exposure of interest since only antipsychotics, anxiolytics, hypnotics/sedatives, and antidepressants were assessed. In another register‐based study in Denmark of patients with dementia initiating antipsychotic treatment, the risk of hospital readmission after combining antipsychotics with benzodiazepines was about 55% higher than with antipsychotic monotherapy after 6 months of follow‐up [24]. However, this study only focused on the combination of antipsychotic therapy and benzodiazepines and was restricted to patients with dementia.

Regarding the outcome of mortality, our results contrast with the Swedish case–control study mentioned above, which observed a strong linear relationship between the number of psychotropic medications in an older population and deaths [26]. The authors found that patients prescribed four psychotropic medications had an approximately 2.5‐fold higher mortality risk than patients taking no psychotropic medications (4 psychotropics vs. 0: OR 2.50; 95% CI: 2.33–2.69). Antipsychotic medications seemed to be particularly strongly associated with mortality, as has been observed in other studies [26, 44, 45]. These inconsistencies in the results may possibly be explained by the fact that the aforementioned study looked primarily at higher‐risk drugs (i.e., antipsychotics, anxiolytics, hypnotics/sedatives), which have a known association with increased mortality. The prevalence of antipsychotic use in our study was relatively small in comparison, which might also explain why we found no significant correlation. Furthermore, we cannot exclude the possibility that we missed such an association due to fewer deaths (16.6%) in our study with a significant pattern of the CI (HR 1.14; CI 1.07 to 1.23).

Our study adds some novel aspects to existing evidence. First, our findings strengthen the existing evidence on increased risk of hospitalization associated with higher numbers of CNS‐active medications, particularly in older multimorbid people for whom evidence hitherto has been weaker than for other populations, such as patients with dementia. Second, to our knowledge, this is the first study to assess the influence of the *number* of CNS‐active medications on DRAs. Our results indicate that the risk of DRA also increases in tandem with increasing numbers of CNS‐active medications. Third, our results extend previous evidence to a broader definition of CNS‐active medications since we not only assessed psychotropic medications (including benzodiazepines, antipsychotics, hypnotics/sedatives, antidepressants) but also CNS‐active medications in general, including also antiparkinsonian, antidementia, and antiepileptic medications.

## 5 Strengths and Limitations

Our study has a number of strengths. First, data quality in our study is high. The data were collected in a prospective, standardized, and almost complete manner within the framework of a trial, and DRAs were adjudicated by an independent committee according to a standardized adjudication guideline [29]. Second, participant loss to follow‐up and withdrawal rates were low, making the risk of bias due to attrition low. Third, we assessed an older multimorbid population, a steadily increasing population of patients often neglected or excluded in clinical studies [46, 47]. Furthermore, there were few exclusion criteria, which supports the generalizability of our findings. Finally, a novel element of the study is the assessment of patient‐reported and patient‐relevant outcomes, namely QoL and functional status, in regard to CNS‐active medications. Our study also has some limitations. First, we considered the use of CNS‐active medication at discharge, whereas changes in medication use—such as with benzodiazepines [48]—during the trial observation time were not taken into account. Hence, our analysis did not differentiate between short‐term and chronic use of CNS‐active medications. We chose to use the prescribed discharge medication list that was documented in the OPERAM trial since we did not have access to data on medication data held in patients' community pharmacies. The discharge prescriptions were used as the best proxy indicator of actual outpatient medication after discharge. Second, our study did not evaluate different medication doses. Third, we had few patients with high‐risk combinations of CNS‐active medications, which can potentially limit conclusions from our assessment of interactions between these medication classes. Fourth, we did not analyze details on the types of DRAs; however, they have previously been described in another study based on the OPERAM population. The most common causes for DRAs were falls/fractures (16%), bleeding (15%) and heart failure exacerbation (13%) [49]. CNS‐active medications were most frequently associated with falls/fractures and confusion/delirium (≥ 5% prevalence) [49]. Finally, our study was limited by the inherent constraints of an observational study. Despite careful identification of possible confounders using established methods and adjusting for these variables in the analysis, the presence of confounding variables by unmeasured or unconsidered factors cannot be excluded. In particular, confounding by indication should be mentioned here. Having a diagnosis of Parkinson's disease, dementia, or epilepsy in itself might be a predictor for adverse outcomes [50, 51], while the CNS‐active medications prescribed are simply markers of these conditions.

Our study results have some important clinical implications. The study's findings should increase clinicians' awareness of the need to carefully weigh the benefits and risks when they prescribe CNS‐active medications and, where possible, to reduce the number of CNS‐active medications in older people with multimorbidity and associated polypharmacy. A potential strategy for avoiding inappropriate prescriptions is to promote and increase access to evidence‐based non‐pharmacologic therapies, such as in the treatment of mild to moderate depression and insomnia [52, 53]. Furthermore, better patient education and physician education may encourage physician consultations to discuss existing CNS‐active medication prescriptions.

For an improved understanding of deprescribing of clinically unnecessary CNS‐active medications in this growing patient population, further studies examining interventions for safe and successful deprescribing are needed. Ultimately, these and other complementary strategies may help to reduce hospital readmissions, which is in the interest of both patients and health care systems worldwide.

## 6 Conclusion

In this prospective study analysis of older adults with multimorbidity and polypharmacy, each additional CNS‐active medication at discharge was associated with an increased risk of all‐cause hospital readmission and DRA at 12 months' follow‐up. While all‐cause mortality was not significantly associated with the number of CNS‐active medications, we observed that the number of CNS‐active medications was associated with lower QoL and reduced ADL functional status. These findings highlight the importance of careful risk–benefit assessment when prescribing CNS‐active medications in this vulnerable population.

## Author Contributions

Conception and design of the study: Carole E. Aubert and Mirah J. Stuber. Analysis and interpretation of data: Mirah J. Stuber. Manuscript drafting: Mirah J. Stuber. Revising the manuscript critically for important intellectual content: Carole E. Aubert. Approval of the version of the manuscript to be published: All authors.

## Disclosure

The Sponsor had no role in the conception/design of the study; data acquisition; analysis and interpretation of data; manuscript drafting; manuscript revision; approval of the manuscript to be published; decision to submit the manuscript.

## Conflicts of Interest

The authors declare no conflicts of interest.

## Supporting information


**Data S1:** Supporting Information.
