# Supplementary material for: Central Nervous System‐Active Medications and Risk of Hospital Readmission in Older Multimorbid Adults
Source: J Am Geriatr Soc. 2025 Aug 29;73(10):3113–22. doi: 10.1111/jgs.70049 (PMC12554834; doi:10.1111/jgs.70049)
Supplement: Supplementary file 1 — Data S1: Supporting Information. [file JGS-73-3113-s001.pdf]

Supplementary File

Central nervous system-active medications and risk of hospital readmission in older multimorbid adults

Contents

eFigure 1: Directed Acyclic Graph (DAG) for identification of confounders for hospital readmission....... 2

eFigure 2: Flow Chart of study population. 3

eFigure 3: Number of patients against number of CNS-medications at discharge. 4

eFigure 4: Subgroup analysis for all-cause hospital readmission. 5

eTable 1: ATC[a] codes of CNS-active medications 6

eTable 2: Additional Sensitivity Analyses....... 8

eTable 3: Hazard ratios and p-values for interactions between different CNS-active medication classes. 10

eFigure 1: Directed Acyclic Graph (DAG) for identification of confounders for hospital readmission

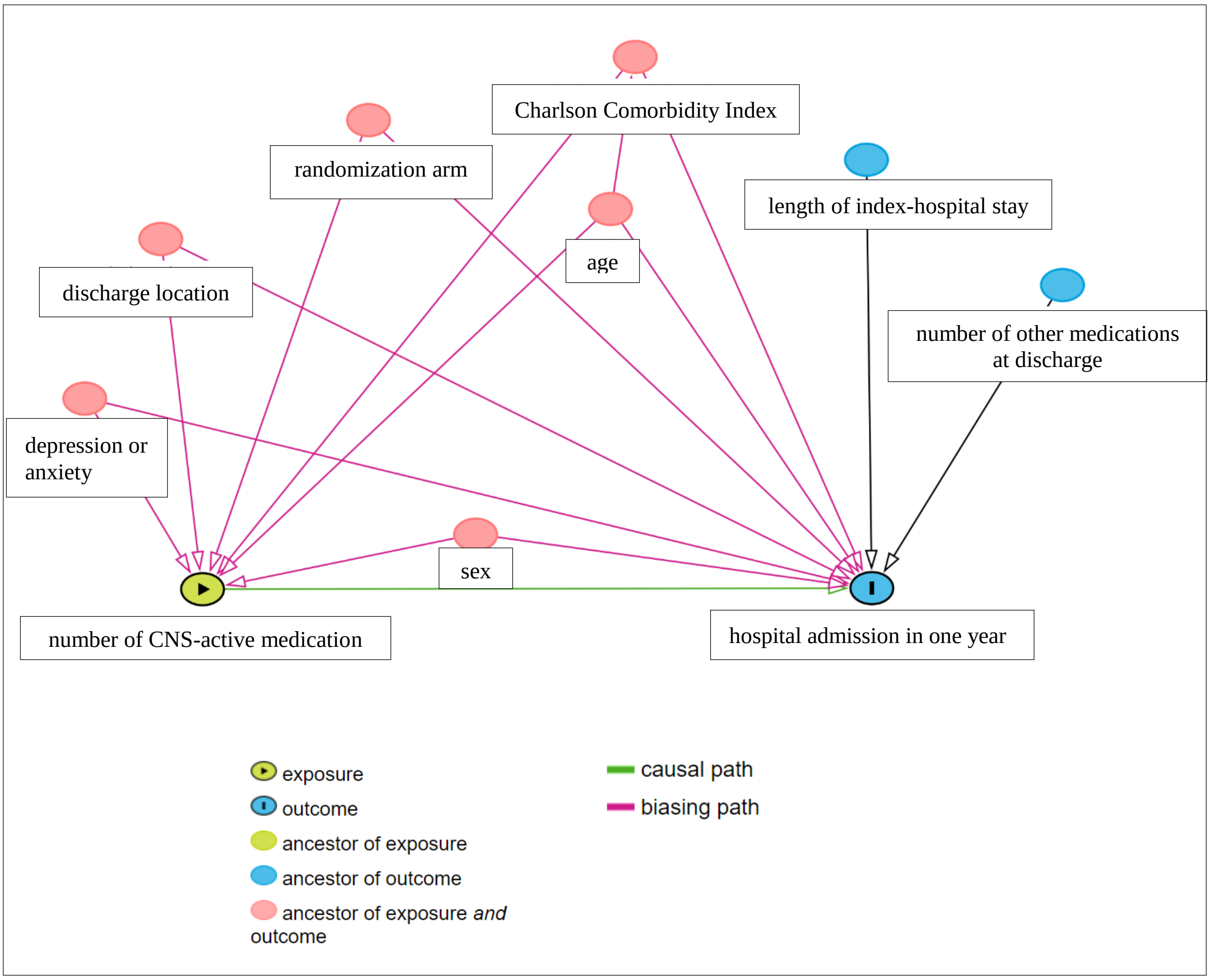

eFigure1. DAG guiding the identification of potential confounders. The graph shows hypothesized relationships between exposure, outcome and covariates for hospital readmission. Confounding variables: presence of depression or anxiety, discharge location (as indirect indicator for location from which patient was admitted to the hospital), randomization arm, Charlson Comorbidity Index, age and sex. Variables, which do not introduce confounding: length of index-hospital stay, number of other medications at discharge.
Abbreviations: DAG, Directed Acyclic Grap; CNS, central nervous system.

eFigure 2: Flow Chart of study population.

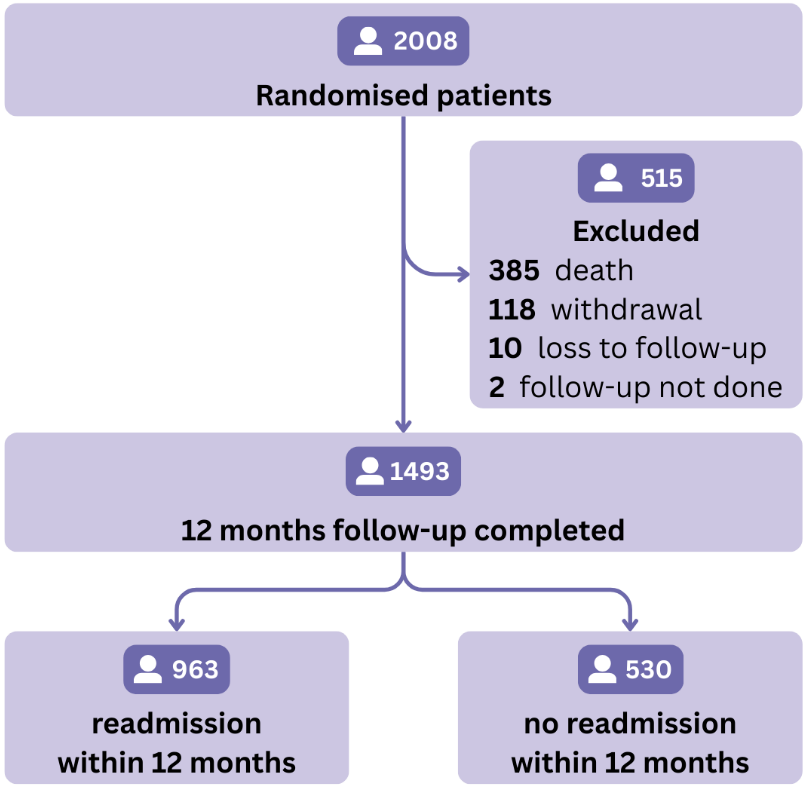

eFigure 3: Number of patients against number of CNS-medications at discharge.

Abbreviations: CNS, central nervous system

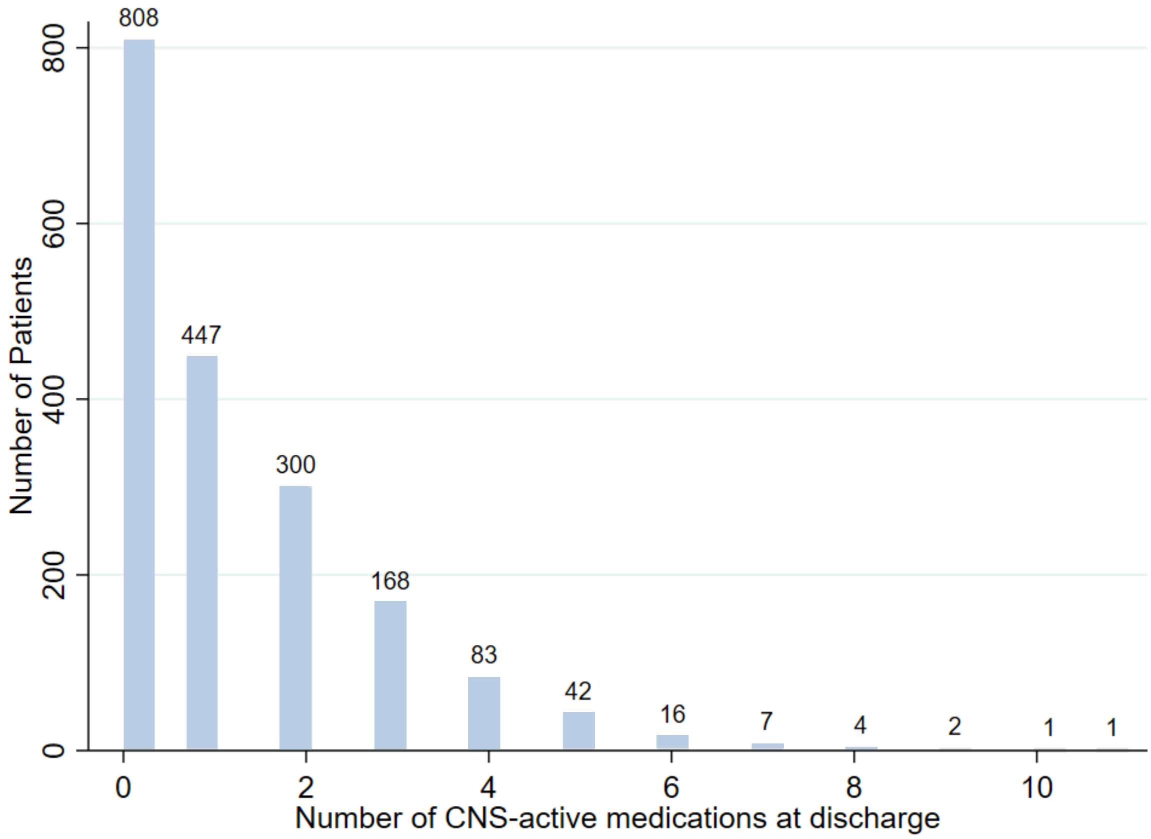

eFigure 4: Subgroup analysis for all-cause hospital readmission.

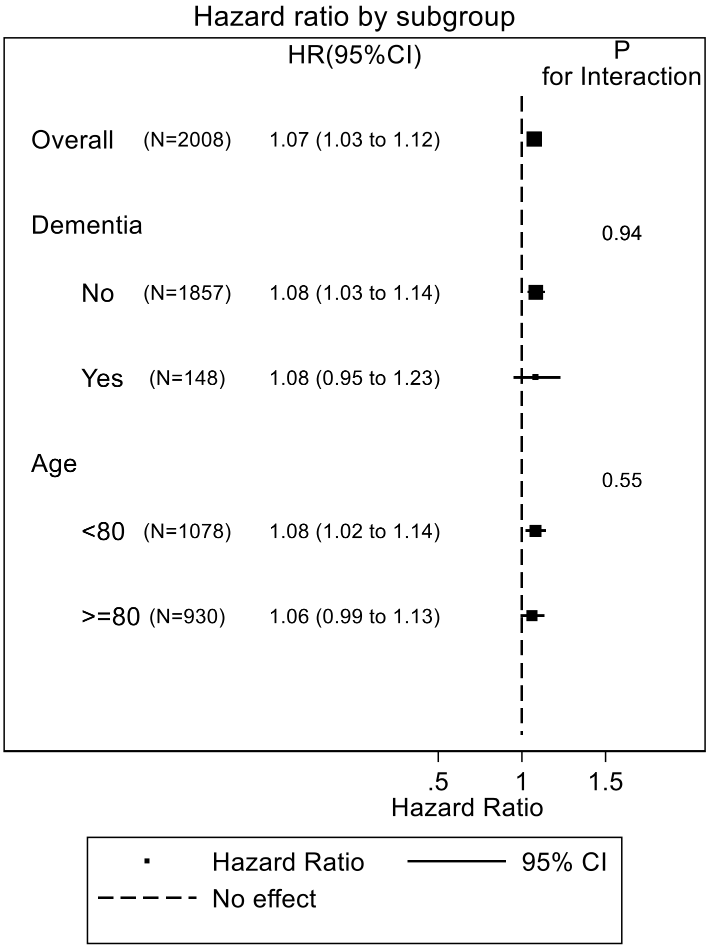

Abbreviations: CI, confidence interval; HR, hazard ratio; N, number; P, P value

eTable 1: ATC[a] codes of CNS-active medications

CNS-active medication class	ATC[a]-Code
ANALGESICS	N02
Opioids	N02A, N02AAxx, N02ABxx, N02ACxx, N02ADxx, N02AExx, N02AFxx, N02AGxx, N02AJxx, N02AXxx
Other Analgesics and Antipyretics	N02B, N02BAxx, N02BBxx, N02BExx
Antimigraine Preparations	N02C, N02CAxx, N02CBxx, N02CCxx, N02CDxx, N02CXxx
ANTIEPILEPTICS	N03, N03A, N03AAxx, N03ABxx, N03ACxx, N03ADxx, N03AExx, N03AFxx, N03AGxx, N03AXxx
ANTIPARKINSON MEDICATIONS	N04, N04A, N04AAxx, N04ABxx, N04ACxx, N04BAxx, N04BBxx, N04BCxx, N04BDxx, N04BXxx, N04C, N04CXxx
PSYCHOLEPTICS	N05
Antipsychotics	N05A, N05AAxx, N05ABxx, N05ACxx, N05ADxx, N05AExx, N05AFxx, N05AGxx, N05AHxx, N05ALxx, N05ANxx, N05AXxx
Anxiolytics	N05B, N05BAxx, N05BBxx, N05BCxx, N05BDxx, N05BExx, N05BXxx
Hypnotics and Sedatives	N05C, N05CAxx, N05CBxx, N05CCxx, N05CDxx, N05CExx, N05CFxx, N05CHxx, N05CMxx, N05CXxx
PSYCHOANALEPTICS	N06
Antidepressants	N06A
SSRI[b]	N06ABxx
TCA[c]	N06AAxx

Other Antidepressants	N06AFxx, N06AGxx, N06AXxx
Psychostimulants, Psycholeptics and Psychoanaleptics in combination	N06B, N06BAxx, N06BCxx, N06BXxx, N06C, N06CAxx
Anti-Dementia Medications	N06D, N06DAxx, N06DXxx
OTHER NERVOUS SYSTEM MEDICATIONS[d]	N07, N07A, N07AAxx, N07ABxx, N07AXxx, N07B, N07BAxx, N07BBxx, N07BCxx, N07C, N07CAxx, N07X, N07XA, N07XXxx

[a] Anatomical Therapeutic Chemical (ATC) classification codes

[b] Selective serotonin reuptake inhibitors

[c] Tricyclic antidepressants

eTable 2: Additional Sensitivity Analyses

**First all-cause hospital readmission within 12 months**		
**Additional adjustments**	**Adjusted hazard ratio (95% CI) for each additional CNS-active medication[a]**	**p-value**
Number of non-CNS active medications	1.07 (1.03 to 1.12)	0.001
Neurological and psychiatric comorbidities (dementia, substance use disorder, psychosis, pain)	1.08 (1.04 to 1.14)	0.001
Smoking, alcohol, education level, living situation	1.07 (1.03 to 1.12)	0.002
Censoring patients at time of deviation from number of CNS-active medications at discharge	1.21 (1.15 to 1.27)	<0.01
**First DRA within 12 months**		
**Additional adjustments**	**Adjusted hazard ratio (95% CI) for each additional CNS-active medication[a]**	**p-value**
Number of non-CNS active medications	1.08 (1.01 to 1.15)	0.019
Neurological and psychiatric comorbidities (dementia, substance use disorder, psychosis, pain)	1.10 (1.03 to 1.18)	0.005
Smoking, alcohol, education level, living situation	1.08 (1.01 to 1.15)	0.026
Censoring patients at time of deviation from number of CNS-active medications at discharge	1.24 (1.15 to 1.34)	<0.01
**Death within 12 months**		
**Additional adjustments**	**Adjusted hazard ratio (95% CI) for each additional CNS-active medication[a]**	**p-value**
Number of non-CNS active medications	1.04 (0.97 to 1.12)	0.253
Neurological and psychiatric comorbidities (dementia, substance use disorder, psychosis, pain)	1.03 (0.95 to 1.11)	0.5
Smoking, alcohol, education level, living situation	1.03 (0.96 to 1.11)	0.403

Censoring patients at time of deviation from number of CNS-active medications at discharge	1.20 (1.11 to 1.30)	<0.01

[a] Initial confounders adjusted for: age (at baseline), sex, discharge location, Charlson Comorbidity Index (at baseline), presence of depression or anxiety (at baseline) and randomization arm. Hazard ratios >1 indicate more events with increasing number of CNS-active medications.

eTable 3: Hazard ratios and p-values for interactions between different CNS-active medication classes.

Class combinations	No (%) of participants	Adjusted hazard ratio (95% CI)[a]	p-value
Opioids and Hypnotics and Sedatives	93 (4.6)	1.26 (0.85 to 1.85)	0.25
Opioids and Anxiolytics	53 (2.6)	1.15 (0.73 to 1.80)	0.55
Antidepressants and Hypnotics and Sedatives	103 (5.1)	0.83 (0.56 to 1.22)	0.35
Antipsychotics and Antidepressants	49 (2.4)	0.88 (0.52 to 1.47)	0.62

[a] Adjusted for age (at baseline), sex, discharge location, Charlson Comorbidity Index (at baseline), presence of depression or anxiety (at baseline) and randomization arm. Hazard ratios >1 indicate more events if medication classes are combined.
